# Ability of Platelet-Derived Extracellular Vesicles to Promote Neutrophil-Endothelial Cell Interactions

**DOI:** 10.1007/s10753-018-0893-5

**Published:** 2018-09-14

**Authors:** Sahithi J. Kuravi, Paul Harrison, George Ed Rainger, Gerard B. Nash

**Affiliations:** 1 Institute of Cardiovascular Sciences, College of Medical and Dental Sciences, University of Birmingham, Birmingham, B15 2TT UK; 2 NIHR Surgical Reconstruction and Microbiology Research Centre, University Hospitals Birmingham NHS Foundation Trust, Birmingham, UK; 3 Institute of Inflammation and Ageing, College of Medical and Dental Sciences, University of Birmingham, Birmingham, B15 2TT UK

**Keywords:** platelets, extracellular vesicles, neutrophils, endothelial cells, cell adhesion

## Abstract

We tested the ability of platelet-derived extracellular vesicles (PEV) to promote adhesion of flowing neutrophils to endothelial cells (EC). PEV were collected from platelets stimulated with collagen-related peptide, and differential centrifugation was used to collect larger vesicles enriched for platelet membrane microvesicles (PMV) or smaller vesicles enriched for platelet exosomes (Pexo). Vesicle binding and resultant activation of neutrophils and EC were assessed by flow cytometry. Flow-based adhesion assays assessed binding of neutrophils directly to deposited vesicles or to EC, after neutrophils or EC had been treated with vesicles. PEV bound efficiently to neutrophils or EC, with resultant upregulation of activation markers. Binding was Ca^++^-dependent and dominantly mediated by CD62P for neutrophils or by integrins for EC. Deposited PEV supported mainly transient attachments of flowing neutrophils through CD62P and some stable adhesion through CXC-chemokines. Neutrophil adhesion to EC was promoted when either cell was pre-treated with PEV, although the effect was less prominent when EC were pre-activated with tumor necrosis factor-α. The pro-adhesive effects on neutrophils could largely be attributed to the larger PMV rather than Pexo. Thus, surface-bound PEV can capture flowing neutrophils, while PEV also activate neutrophils and EC to promote interactions. PEV may potentiate inflammatory responses after tissue injury.

## INTRODUCTION

Platelet extracellular vesicles (PEV) are sub-micron particles released from activated, apoptotic, or stored platelets [1–3]. They form a major portion of the normal EV population found in the plasma in the circulation [4], with raised numbers in various pathologies, including trauma, venous thromboembolism, rheumatoid arthritis, ischemia, and uremia (reviewed in [5]). PEV carry a diverse cargo of proteins, with constituents depending on the platelet activation process [6, 7]. Their content can be transferred to other cells through pathways involving endocytosis, macropinocytosis, phagocytosis, and lipid raft-mediated fusion and internalization (reviewed by Mulcahy et al. [8]). PEV can thus mediate crosstalk between cells by transferring active agents, for instance influencing haemostatic and inflammatory responses [9–11].

The role(s) of PEV in inflammation are facilitated by their capacity to bind to leukocytes and endothelial cells (EC) [9, 12, 13]. Uptake and bioactivity of PEV may depend on the binding proteins expressed on the PEV and recipient cells and on specific agents in their cargo. Phosphatidyl serine is typically exposed on the PEV surface, along with adhesive glycoproteins including GP1b (CD42b), GPIIbIIIa (αIIbβ3-integrin; CD41CD61), β1-integrin (CD29), and P-selectin (CD62P), while cargo carried includes proinflammatory cytokines and chemokines such as interleukin (IL)-1β, IL-8 (CXCL8), IL-6, and RANTES (CCL5) [13–21]. The effector role of PEV in inflammation may arise from promotion of leukocyte-endothelial interactions. EV uptake has been shown to upregulate activation markers, such as CD11b on neutrophils (polymorphonuclear cells, PMN) and CD54/ICAM-1 on EC, and increase leukocyte adhesion to EC *in vitro* and *in vivo* [13, 15, 22, 23]. However, the mechanisms by which PEV might bind to PMN and EC, and the means by which PEV might promote PMN recruitment from flow are not well-defined to date.

PEV are not homogenous and can be broadly classified into plasma membrane-derived microvesicles (PMV; diameter about 100–1000 nm) and platelet exosomes (Pexo; about 40–100 nm) [24–26]. Pexo are intracellular nanovesicles released through exocytosis [26], while PMV are shed from the plasma membrane and usually characterized by exposed phosphatidylserine [24–27]. Proteomic studies have analyzed the different protein contents of these PEV fractions [28–31]. In addition to their difference in size, PMV and Pexo were shown to have complicated molecular profiles with some overlap, which may impart diverse functions in physiological and pathological processes [7, 31–34]. However, studies so far have usually characterized the effects of total PEV populations in inflammatory processes, with only the occasional study of the role of PMV or Pexo separately [26].

Here, we aimed to investigate the ability of PEV to promote interactions when PMN were perfused over EC. The EC and/or PMN were treated with PEV before the PMN were perfused, while in some studies, a surface was coated with PEV to test the direct ability of deposited PEV to capture PMN. Cell-binding kinetics for PEV and consequent upregulation of activation markers on PMN and EC were also studied, as were the effects of inhibitors of adhesion molecules and chemokines likely to influence interactions between the different vesicles and cells. Some studies were repeated for PEV that had been centrifugally fractionated to enrich for PMV or Pexo. In this way, we aimed to gain insight into the potential pro-inflammatory roles of PEV acting on PMN and/or EC, and mechanism that would underlie them.

## METHODS

### Antibodies and Inhibitors

Antibodies used for label and immunofluorescence studies were as follows: FITC-conjugated anti-CD41 (clone 5B12, Dako, UK), anti-CD42b (clone HIP1, BD Pharmingen, UK), anti-CD62P (P-selectin, clone AK-4, BD Pharmingen, UK), and anti-CD106 (VCAM-1, clone 51-10C9, BD Pharmingen, UK); PE conjugated anti-CD62E (E-selectin, clone 1.2B6, Sigma-Aldrich,UK), anti-CD31 (clone 9G11, R&D systems, UK), and anti-CD11b (α_m_-integrin, clone 2LPM19c, Dako, UK); APC-conjugated anti-CD54 (ICAM-1, clone HA58, BD Pharmingen, UK) and anti-CD144 (VE-Cadherin, clone 16B1, ebioscience, UK); and control FITC- and PE-conjugated IgG1 (Dako, UK) or APC-conjugated IgG1 (BD Pharmingen, UK). All were used at suppliers’ recommended dilutions.

Function blocking antibodies were as follows: anti-CD541 (20 μg/ml, clone 8.4A6 Sigma-Aldrich, UK), anti-CD106 (20 μg/ml, clone BBIG-V1, R&D Systems, UK), anti-CD62E (20 μg/ml, clone H4/18, BD Pharmingen, UK), anti-CD11b (10 μg/ml, clone ICRF44, Ebioscience, UK), anti-CXCL5 (ENA-78, 10 μg/ml, clone 33160, R&D systems, UK), anti-CXCR1 & 2 (4 μg/ml, clones 501 & 19, respectively, Abcam, UK), anti-CD42b (GP1b, 10 μg/ml, clone SZ2, Beckman Coulter, UK), anti-CD62P (10 μg/ml, clone G1, Ancell), anti-CD29 (β_1_-integrin, 10 μg/ml, Mab 13, BD Pharmingen, UK), CD61 (β_3_-integrin, 10 μg/ml, clone SZ21, Beckman Coulter, UK), anti-CD51 (α_v_-integrin, 10 μg/ml, clone L230, Enzo Lifesciences, UK), and anti-CD41/CD61 (GPIIb/IIIa, α_IIB_β_3_-integrin 10 μg/ml, clone c7E3, Janssen Biologics BV, Netherlands). Additional inhibitors were the peptide Arg-Gly-Asp-Ser (RGDS, 0.5 mM, Sigma-Aldrich, UK) and EDTA (10 mM, Sigma-Aldrich, UK).

### Isolation and Label of Platelets and Generation of PEV

Blood samples were obtained from healthy, adult volunteers, with written, informed consent and approval from the University of Birmingham Local Ethical Review Committee. Studies were performed in accordance with the ethical standards as laid down in the 1964 Declaration of Helsinki. Venous blood was collected into tubes with anticoagulant citrate phosphate dextrose adenine solution (CPDA, 1:9 with blood *v*/*v*, Sigma-Aldrich, UK). Platelet isolation was as described previously [35]. Blood was centrifuged at 250 *g* for 15 min at RT with added theophylline (7 mM, Sigma-Aldrich, UK) to maintain a resting state. The supernatant platelet-rich plasma (PRP) was collected into a polypropylene tube and diluted with phosphate buffered saline without Ca/Mg (PBS, Sigma) with 0.15% bovine serum albumin (BSA, Sigma) and 7-mM theophylline (PBSA-T) and centrifuged at 800 *g* for 15 min to pellet the resting platelets. Supernatant was discarded and pelleted platelets were resuspended in PBS with BSA. Platelet concentration was measured with a Coulter T-540 and adjusted to 3 × 10^8^ platelets/ml in PBS with BSA, with Ca/Mg (PBSA), or in PBSA-T if they were to be labeled.

For labeling, platelets in PBSA-T were incubated with FITC-conjugated anti-CD41, anti-CD42b, anti-CD62P, or IgG1 control for 30 min in the dark at room temperature. Alternatively, platelets were stained with the cell membrane label PKH67 (Sigma) according to the manufacturer’s instructions. The platelets were washed free of unbound antibody or dye by resuspending in 15mls of PBSA-T without Ca/Mg and centrifuged at 800 *g* for 15 min to pellet the platelets. The pellet was resuspended in PBSA at a concentration of 3 × 10^8^ platelets/ml.

PEV were generated using labeled or unlabeled platelets at 3 × 10^8^/ml as required. Initially, platelets were incubated for 30 min at 37 °C with 1 μg/ml of crosslinked collagen related peptide (CRP-XL) (from Professor Farndale, University of Cambridge, UK) or thrombin receptor-activating peptide (TRAP, 10 μM, Bachem AG, Switzerland) or in the absence of agonist as a control. Platelets were removed by centrifugation at 2000 *g* for 20 min followed by transfer of supernatant to a fresh Eppendorf, which was centrifuged for 2 min at 13,000 *g* to remove residual platelets [36, 37]. The supernatant contained PEV, which can be sub-classified into platelet microvesicles (PMV) and platelet exosomes (Pexo). To enrich for PMV, 1-ml supernatant was transferred into a new tube and centrifuged for 45 min at 16,500 *g* at 4 °C. The pelleted larger vesicles were collected and resuspended in 1-ml PBSA. The supernatant was then transferred to a fresh tube and subjected to centrifugation for 1 h at 100,000 *g* at 4 °C. To obtain enriched Pexo, the supernatant was discarded and the vesicles resuspended in 1-ml PBSA. PEV suspensions or other prepared samples were frozen at − 20 °C until further use.

### Characterization of PEV by Nanoparticle Tracking Analysis (NTA) and Flow Cytometry

Size distribution and concentration of PEV and centrifugal fractions were determined using a Nanosight LM10 (Malvern Instruments, UK) equipped with NTA software 2.2 as described [38]. The minimum size detected was ~ 50 nm. Samples were diluted with filtered PBS to achieve an optimum particle concentration of 10^8^–10^9^/ml. Three hundred microliters of sample was introduced into a chamber held on a light microscope, which was illuminated by laser at an angle to the optical axis of the microscope. A digital camera attached to the microscope visualized the scattered light from particles, and images were captured at a rate of 30/s for 60 s. Individual particles were counted and tracked, and their Brownian motion analyzed to yield their velocity and hence diameter. The recording yielded the frequency distribution of vesicle sizes and an estimate of the total number of vesicles/ml.

PEV derived from labeled platelets were analyzed on a BD Accuri C6 flow cytometer (BD, Oxford, UK). The gating window for counting PEV and discriminating against background noise was set using forward and side scatter plots for Megamix fluorescent polystyrene beads (BioCytex, France) of diameters 500 nm, 900 nm, and 3 μm as described [38]. EV counts were taken from the gate that included 500- and 900-nm megamix beads. Samples were analyzed at a low flow rate (14 μl/min) until 2000 positive events were collected in the EV gate. The total EV numbers were acquired from counts in the microvesicle gate defined as above, for a known sample volume. The label-specific counts were obtained from fluorescent signals for the different labels analyzed one at a time, for particles that fell in light scatter gate and were positively labeled compared to fluorescent isotype controls.

The PEV counts and particle diameters analyzed by NTA, and the counts analyzed by flow cytometry were similar for platelets treated with TRAP of CRP-XL (data not shown). Values for CRP-XL are reported below and for further studies CRP-XL was used, to avoid transfer of a stimulatory agent (TRAP) along with PEV when treating PMN and/or EC.

### Isolation of PMN and Human Umbilical Vein Endothelial Cells

PMN were isolated from venous blood from consented, healthy, adult volunteers collected into EDTA tubes (Sarstedt Ltd., Leicester, UK). PMN were isolated by overlaying blood on density gradients of histopaque 1077 over histopaque 1119 (Sigma-Aldrich, UK) as described [39]. Collected PMN were washed using PBSA, counted using a Cellometer auto T4 cell counter (Nexcelom Bioscience Ltd., Manchester, UK), and adjusted to 10^6^ cells/ml in PBSA.

Human umbilical vein endothelial cells (HUVEC) were isolated from umbilical cords by collagenase treatment as described previously [40]. Umbilical cords were obtained from the Human Biomaterials Resource Centre (University of Birmingham) after informed consent. HUVEC were cultured at 37 °C with 5% CO_2_ in M199 supplemented with 20% fetal calf serum, 10-ng/mL epidermal growth factor, 35-μg/mL gentamicin, 1-μg/ml hydrocortisone (Sigma-Aldrich, UK), and 2.5-μg/mL amphotericin B (Life Technologies, CA). Confluent primary HUVEC were detached with trypsin/EDTA (Sigma), washed, and reseeded into 24 well plates (Corning, UK) or into flow channels (Ibidi u-Slide VI (0.4), Thistle Scientific, UK) for studies of vesicle uptake or flow-based adhesion experiments, respectively.

### Binding Kinetics of PEV to PMN or HUVEC

Isolated PMN were incubated with CD41-labeled PEV or PKH-labeled PEV at a concentration of 10^9^ PEV/ml in PBSA in a test tube placed on roller mixer at 37 °C. At chosen times, PMN were washed from unbound PEV by centrifuging at 400 *g* for 5 min and the PMN pellet was labeled with PE-conjugated anti-CD11b for 30 min in the dark at room temperature. CD11b labeled PMN were washed from unbound antibody by pelleting as described above and were fixed in 100-μl 2% (*w*/*v*) formaldehyde. Fixed PMN were analyzed using a BD Accuri C6 flow cytometer to determine the percentage positively stained with PEV label and CD11b. Alternatively, images were captured using a Zeiss LSM780 confocal microscope. To investigate the mechanism of binding of PEV, monoclonal antibodies against CD11b, GP1b, CD62P, CXCR3, GPIIb3a or control IgG1, or RGDS, or EDTA were added either to PMN or PEV prior to their mixing and incubation.

Confluent HUVEC grown in 24 well plates were incubated with CD41-labeled PEV at a concentration of 10^9^ PEV/well in PBSA. At chosen times, HUVEC were washed from unbound PEV using PBSA and the cells were labeled for EC marker APC-conjugated CD144 for 30 min at 37 °C. The labeled cells were subjected to Accutase treatment (Stem pro dissociation reagent, Gibco, UK) to dissociate cells into suspension and centrifuged at 400 *g* for 5 min to remove enzyme activity and unbound antibody. Labeled cells were fixed in 100-μl 2% (*w*/*v*) formaldehyde and analyzed using a BD Accuri C6 flow cytometer to determine the percentage positively stained with PEV label and CD 144. Alternatively, images were captured using a Zeiss LSM780 confocal microscope. To investigate the mechanism of binding of PEV, monoclonal antibodies against CD51, CD29, CD61, GPIIbIIIa, CD62P or control IgG1, or RGDS or EDTA were added either to HUVEC or PEV prior to their mixing and incubation.

### Detection of HUVEC or PMN Activation

Surface expression of HUVEC adhesion molecules was assessed by label with fluorescent antibodies against ICAM-1, VCAM-1, or E-selectin, followed by analysis by a BD Accuri C6 flow cytometer to measure percentage positive and median fluorescence intensity (MFI). PMN activation was assessed from the MFI for CD11b label, using the same samples as those described above for assessing percentage positive for PEV markers.

### Flow-Based PMN Adhesion Assays

To assess the direct ability of PEV on a surface to capture PMN under flow, PEV were incubated for 1 h at 37 °C in microslides (glass capillaries with rectangular cross-section 300 μm × 3 mm and 50-mm long; Camlab Ltd., Cambridge, UK) that had been treated with aminopropyltriethoxysilane (Sigma-Aldrich, UK) as described [39]. To assess adhesion of PMN to EC, HUVEC sub-cultured into ibidi μ-slides were treated with PEV added to culture medium 1:1 *v*/*v*, for 1, 4, or 24 h. In some experiments, HUVEC were treated at the same time with 1, 5, or 100-U/ml tumor necrosis factor-α (TNF; Sigma) for 4 h.

PMN adhesion was analyzed in a flow-based assay as described previously [35, 40]. Briefly, ibidi μ-slides or microslides were placed on the stage of a phase-contrast video microscope at 37 °C and connected to a Harvard syringe pump set to a flow rate equivalent to a wall shear stress of 0.025, 0.05, or 0.1 Pa. The other end was connected *via* an electronic valve to allow perfusion of PBSA or PMN suspended in PBSA. After flushing with PBSA, PMN were perfused for 4 min followed by washout of non-adherent cells with PBSA. A series of video recordings were made along the centerlines of the flow chambers and analyzed offline using Image Pro software (Image-ProPlus, UK). After washout, the number of adherent PMN was counted from the images and converted to PMN/mm^2^/10^6^ perfused based on the known field size, sample concentration, and flow rate. To analyze transient capture events during inflow, PMN that were visible and formed short-lived attachments on the PEV-coated surface during perfusion were counted in a fixed period, converted to number/mm^2^/min, and multiplied by the area of the chamber to obtain a value for total cells captured from flow/min. In some experiments, PMN were pre-treated with PEV at 10^9^/ml for 30 min at 37 °C before perfusion over surfaces in the continued presence of the PEV.

In some experiments, PEV deposited on the microslides were pre-treated with blocking antibodies against CD62P or CXCL5 for 30 min prior to the perfusion of PMN. Alternatively PMN were treated with antibodies against CXCR1 or CXCR2 for 30 min at 37 °C. PMN were washed by centrifugation at 400 *g* for 5 min prior to the perfusion over PEV or HUVEC. In others, HUVEC were pretreated with blocking antibodies against ICAM-1, VCAM-1, E-selectin, or CXCL5 for 4 h along with PEV incubation at 37 °C.

### Chemokine Detection in PEV Supernatant

Supernatants containing PEV or centrifugal fractions were screened for soluble mediators using a multiplex bead immunoassay (R&D Systems, UK), with or without pre-filtration through 0.2-μm pore filters or 100-kD molecular cutoff filters (Millipore, UK). The immunoassay measured levels of CXCL8, CCL5, CXCL5, and CXCL1 (growth-related oncogene, GRO-α).

### Statistical Analysis

Variation between multiple treatments was evaluated using analysis of variance (ANOVA), followed where appropriate with post hoc comparisons to control by Dunnett test or between conditions by Bonferroni test. Effects of single treatments were analyzed by paired t test compared to controls. Data is expressed as the mean ± SEM, and a *p* value of < 0.05 was considered statistically significant.

## RESULTS

### Characterization of Vesicles Derived from Activated Platelets

**Fig. 1:**
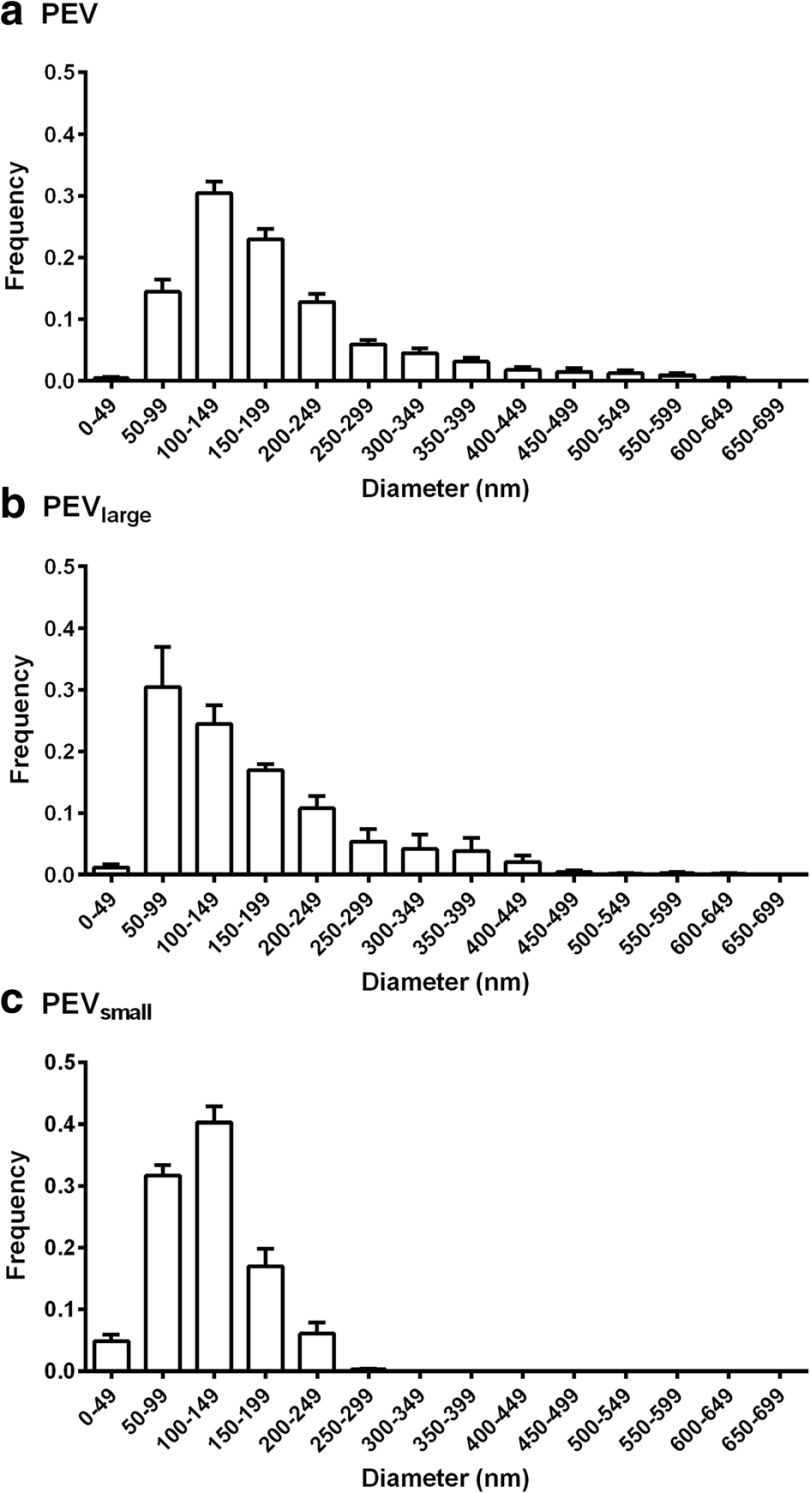
Vesicle diameter analyzed by NTA. Frequency distributions of particle diameter measured for: (**a**) PEV, *n* = 19, (**b**) PEV_large_ enriched for PMV, *n* = 5, (**c**) PEV_small_ enriched for Pexo, *n* = 5. Data are pooled from the n separate samples of vesicles prepared from platelets activated by CRP-XL.

PEV were generated from platelets suspended at 3 × 10^8^/ml stimulated by CRP-XL and isolated by double centrifugation. The total PEV count analyzed by NTA was 1.25 ± 0.34 × 10^10^ particles/ml (mean ± SEM; *n* = 19). The size distribution of PEV is shown in Fig. 1a; diameter varied between 50 and 700 nm and the mode was between 100 and 150 nm (Fig. 1a). Flow cytometry counted 1.5 ± × 10^6^ particles/ml (mean ± SEM; *n* = 8) in a gate which incorporating 500- and 900-nm calibration beads. PEV from platelets labeled with fluorescent antibodies and analyzed by flow cytometry proved to be 70 ± 3% positive for CD41, 37 ± 3% positive for CD42b, and only 18 ± 4% positive for CD62P (mean ± SEM for at least six experiments). Subsequent studies of binding of PEV to cells utilized CD41 as the label. In some experiments, where platelets were labeled with PKH67, 91 ± 4% of PEV were positively labeled (mean ± SEM, *n* = 5).

We also analyzed the size of PEV enriched for platelet microvesicles by centrifuging the PEV for 45 min at 16,500 *g* and PEV enriched for platelet exosomes collected by centrifuging the supernatant from that preparation for 1 h at 100,000 *g*. Since the exosome-rich fraction could not be shown to be exclusively of endosomal origin, the two fractions were labeled as large or small PEV (PEV_large_ and PEV_small_, respectively). NTA showed that the former contained a larger proportion of particles extending to a larger diameter ~ 500 nm, and the latter were restricted to particles < 250 nm (Fig. 1b, c). There was not a clear separation into two, non-overlapping populations with respect to size.

### Characterization of Binding of PEV to PMN

**Fig. 2:**
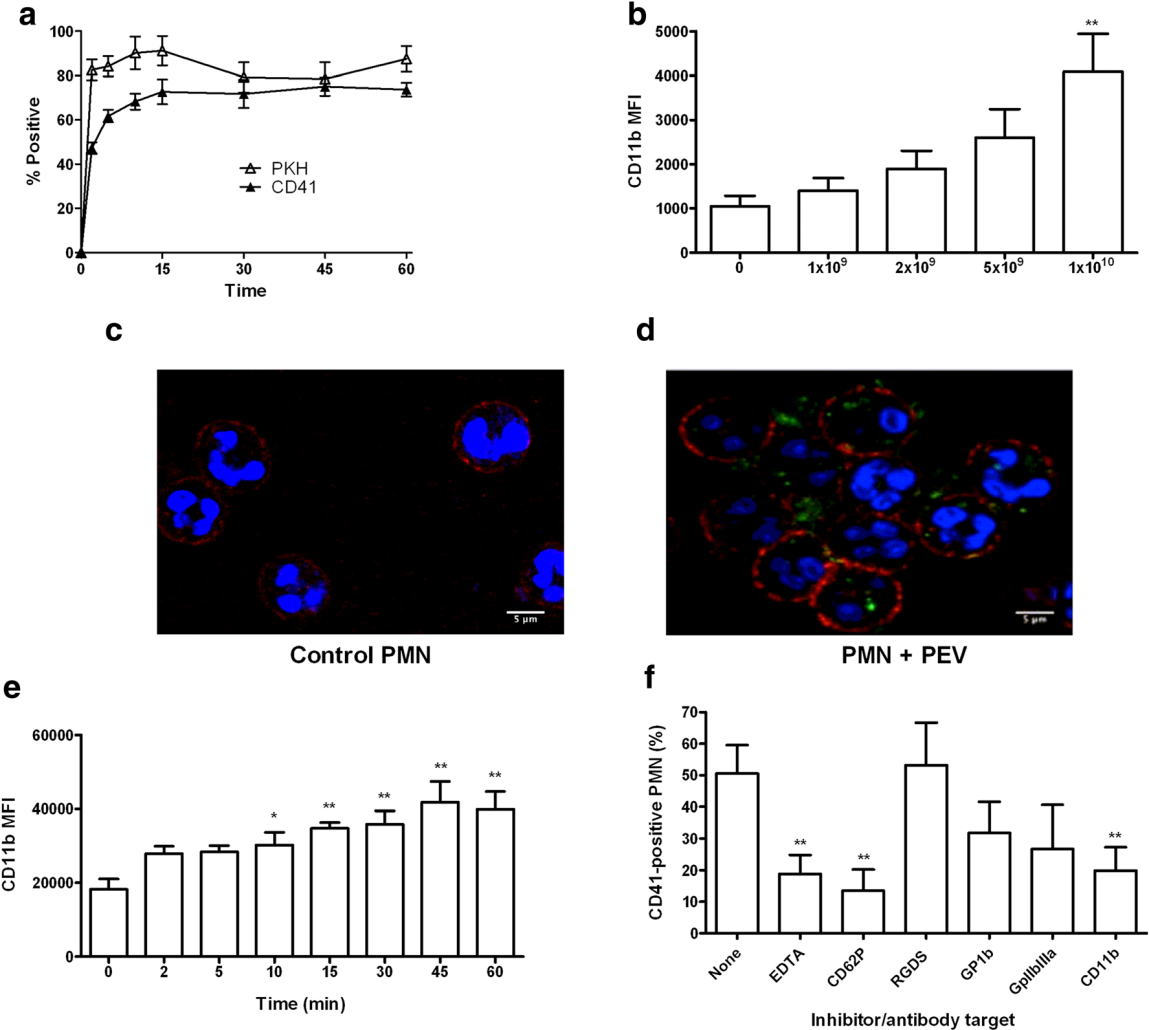
Characterization of binding of PEV to PMN. (**a**) Percentage of PMN positively stained with PEV from platelets labeled with anti-CD41 (*n* = 6) or PKH67 (*n* = 4) for different incubation times. One-way ANOVA showed a significant effect of time for each treatment (both *p* < 0.01); *p* < 0.01 compared to zero time at all subsequent times by Dunnett test. (**b**) Effect of PEV concentration on MFI of PMN incubated for 30 min with PEV (*n* = 3). One-way ANOVA showed a significant effect of concentration (*p* < 0.01). ** = *p* < 0.01 compared to zero PEV by Dunnett test. (**c**), (**d**) Representative confocal microscope images of PMN either untreated (**c**) (control) or incubated (**d**) for 30 min with PEV from platelets labeled with FITC-conjugated anti-CD41 (green). PMN were also labeled with anti-CD11b (red) and propidium iodide (blue). (**e**) Change in surface expression of CD11b on PMN with time during incubation with PEV; MFI determined by flow cytometry (*n* = 6). One-way ANOVA showed a significant effect of time (*p* < 0.01). * = *p* < 0.05, ** = *p* < 0.01 compared to zero time by Dunnett test. (**f**) Effects of inhibitory agents or function-blocking antibodies on labeling of PMN with PEV from platelets labeled with anti-CD41. PMN were incubated with PEV for 30 min in the presence of EDTA (*n* = 7), anti-CD62P (*n* = 9), RGDS (*n* = 7), anti-GP1b (*n* = 10), anti-GPIIbIIIa (*n* = 5), or anti-CD11b (*n* = 9). One-way ANOVA showed a significant effect of inhibitory treatments (*p* < 0.01). ** = *p* < 0.01 compared to none by Dunnett test. All data are mean ± SEM from n experiments.

We mixed isolated PMN with PEV pre-labeled with antibody against CD41 or dye PKH67, in the continued presence of labeled antibody to CD11b (α_m_-integrin), to assess the kinetics of uptake of PEV label by the PMN and their level of expression of CD11b by flow cytometry. Within 2 min, a high proportion of PMN were positive for CD41 or PKH67, with further slower increase over about 15 min to ~ 80% positive (Fig. 2a). There was initially a higher proportion positive with PKH67, but final values were similar for CD41. Further studies used this more specific label unless stated otherwise. Examining the effect of varying the number of PEV added, after 30-min incubation, the proportion of PMN positively labeled was insensitive to the number added between 10^9^ and 10^10^ (data not shown), but the MFI increased steadily over this range (Fig. 2b). Confocal microscopy indicated that intracellular localization of many PEV within the PMN occurred by 30 min (Fig. 2c, d). Interestingly, during the binding of PEV to PMN, there was also a steady increase in the level of CD11b expressed on the surface of the PMN (Fig. 2e). There was approximately 50% increase in MFI of CD11b expression by 2 min, with the increase becoming 90% by 15 min. Thus, PEV binding appeared to activate the PMN.

Next, we investigated the binding mechanisms of PEV to PMN by using blocking reagents. Pretreatment of PMN with EDTA or antibody against CD62P significantly reduced PEV binding by ~ 60 or ~70%, respectively (Fig. 2f). RGDS peptide did not reduce binding. Antibodies against GPIb or GPIIbIIIa tended to reduce binding but not significantly (Fig. 2f), but a blocking antibody against αm-integrin, CD11b (distinct from that used for fluorescence label) did significantly inhibit binding. Thus, PEV-PMN interactions required Ca^+2/^Mg^++^, consistent with the binding requirements of CD62P and CD11b.

### Characterization of Binding of PEV to HUVEC

**Fig. 3:**
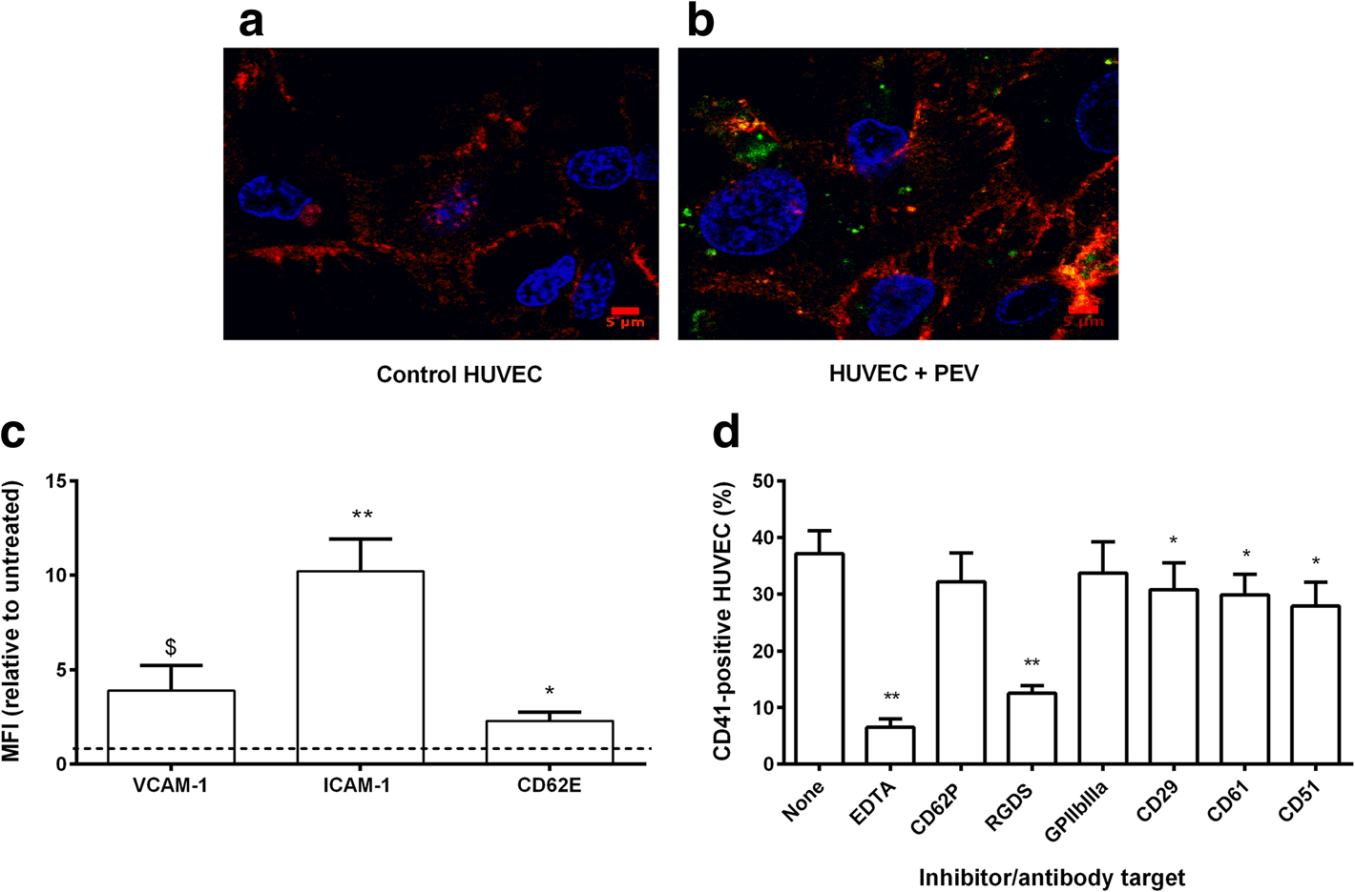
Characterization of binding of PEV to HUVEC. (**a**), (**b**) Representative confocal microscopy images of HUVEC either untreated (**a**) (control) or incubated (**b**) for 4 h with PEV from platelets labeled with FITC-conjugated anti-CD41 (green). HUVEC were also labeled with anti-CD144 (red) and propidium iodide (blue). (**c**) Changes in surface expression of VCAM-1, ICAM-1, or CD62E on HUVEC after incubation with PEV for 4 h (determined by MFI relative to untreated control) (*n* = 6). $ = *p* = 0.054, * = *p* < 0.05 ** = *p* < 0.001 compared to control by paired t test. (**d**) Effects of inhibitory agents or function-blocking antibodies on labeling of HUVEC with PEV from platelets labeled with anti-CD41. HUVEC were incubated with PEV for 4 h in the presence of EDTA (*n* = 7), anti-CD62P (*n* = 10), RGDS (*n* = 7), anti GPIIbIIIa (*n* = 8), anti-CD29 (*n* = 11), anti-CD61 (*n* = 11), or anti-CD51 (*n* = 10). One-way ANOVA showed a significant effect of inhibitory treatments (*p* < 0.01). * = *p* < 0.05, ** = *p* < 0.01 compared to none by Dunnett test. All data are mean ± SEM from n experiments.

Binding of PEV to HUVEC was characterized by the uptake of CD41+ PEV and flow cytometry. Here, we found that 43 ± 6.5% of HUVEC had bound labeled PEV within 1 h, increasing to 51 ± 4% by 4 h and remaining constant at ~ 50% at 24 h (data are mean ± SEM, *n* = 7). Confocal images indicate the intracellular localization of the PEV in HUVEC monolayer at 4 h (Fig. 3a, b). We also analyzed activation of HUVEC in response to PEV binding, based on expression of adhesion molecules typically up-regulated in inflammation. We detected that PEV induced significant increase in surface expression of ICAM-1 and E-selectin by 4 h, and an increase in VCAM-1 that did not reach significance (*p* = 0.085) (Fig. 3c).

Mechanisms of PEV binding to HUVEC were tested using blocking reagents and antibodies. EDTA significantly reduced PEV binding by ~ 80%, but blockade of P-selectin did not reduce binding (Fig. 3d). RGDS reduced binding by nearly 70%. Blocking GPIIbIIIa had little effect, while blocking β1-, β3-, or α_ν_-integrins (CD21, CD61, CD51, respectively) each caused a significant reduction of binding by about 20% (Fig. 3c). These results suggest that Ca^+2/^Mg^++^-dependent integrin interactions played a major role in PEV binding to HUVEC.

### Ability of PEV to Directly Recruit Flowing PMN

**Fig. 4:**
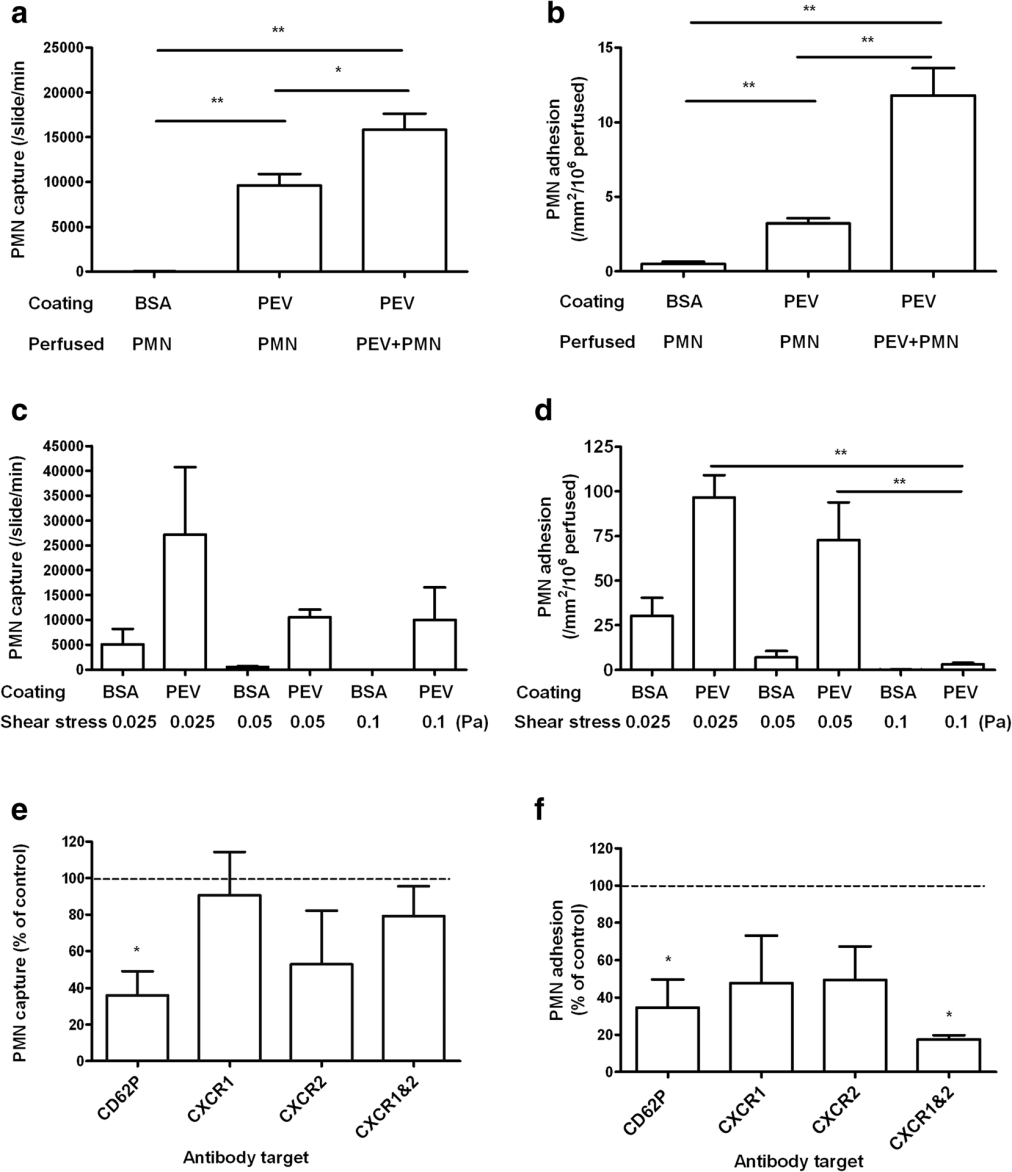
Ability of PEV to directly support PMN recruitment from flow. (**a**) Comparison of the number of transient capture events for PMN perfused at a wall shear stress of 0.1 Pa over surfaces coated with BSA or PEV. Neutrophils were perfused alone (*n* = 13) or with PEV added to the cell suspension (*n* = 5). (**b**) Stably adherent PMN on surfaces coated with BSA or PEV, after perfusion under the same conditions, and washout of non-adherent cells. One-way ANOVA showed a significant effect of treatment combinations on capture events and stable adhesion (*p* < 0.01 in each case). * = *p* < 0.01, ** = *p* < 0.01 by Bonferroni test. (**c**), (**d**) Effect of varying wall shear stress on transient capture events (**c**) or stable adhesion (**d**) for PMN perfused over surfaces coated with BSA or PEV (*n* = 6). Two-way ANOVA showed a significant effect of surface treatment (*p* < 0.01) but not shear stress on capture events and significant effects of surface treatment and of shear stress on stable adhesion (*p* < 0.01 in each case). ** = *p* < 0.01 by Bonferroni test. (**e)**, (**f**) Effects of function-blocking antibodies on transient capture events (**e**) or stable adhesion (**f**) of PMN perfused over surfaces coated with PEV. Coated surfaces were pre-treated with anti-CD62P (*n* = 4), or PMN were pre-treated with anti-CXCR1 (*n* = 4), anti CXCR2 (*n* = 4), or both (*n* = 3). Data are shown as percent of values without antibody. Not all treatments were used in all experiments, but untreated controls were tested in each experiment. * = *p* < 0.05 compared to control by paired *t* test. All data are mean ± from *n* experiments.

To test whether PEV could directly support recruitment of PMN on a surface, glass microslides were coated with PEV (1 × 10^10^/ml) and perfused with PMN at a wall shear stress of 0.1 Pa. Video observations demonstrated multiple short-lived adhesive capture events (> 9500 PMN captured from flow/slide/min), compared to none on BSA coating (Fig. 4a). The duration of transient attachments was 0.25 ± 0.03 s (mean ± SEM; *n* = 34 cells). We also observed further ~ 50% increase in PMN capture from flow when PMN were pretreated and perfused along with PEV over the PEV-treated surface (Fig. 4a). The adhesion was again transient, with PMN hopping across the surface with a capture time increased to 0.4 ± 0.07 s (mean ± SEM *n* = 19 cells). Only a small number of captured PMN adhered firmly, although this number did increase when PMN were pretreated with PEV (Fig. 4b). In two experiments, increasing the number of PEV used to coat the microslides from 1 × 10^10^/ml to 2.5 × 10^10^/ml also increased the number of short attachments (from 4550 to 18,700 PMN/slide/min) and especially the number firmly adherent (from 4 to 116 PMN/mm^2^/10^6^ perfused) (means, *n* = 2). We also tested the effect of perfusing PMN at lower shear stresses of 0.05 or 0.025 Pa. The rate of capture events was not significantly altered, although still higher for surfaces coated with PEV (Fig. 4c). However, there were significantly more stably adherent PMN at the lower stresses Fig. 4d).

Examining the mechanisms involved in direct recruitment of PMN by PEV, blockade of CD62P on PEV caused > 60% inhibition of capture events and stable adhesion (Fig. 4e, f). Blocking CXCR1, CXCR2, or both had variable and non-significant effects on capture. While the individual agents also tended to reduce stable adhesion, only the combination reached a statistically significant effect, with adhesion reduced by > 80%.

**Fig. 5:**
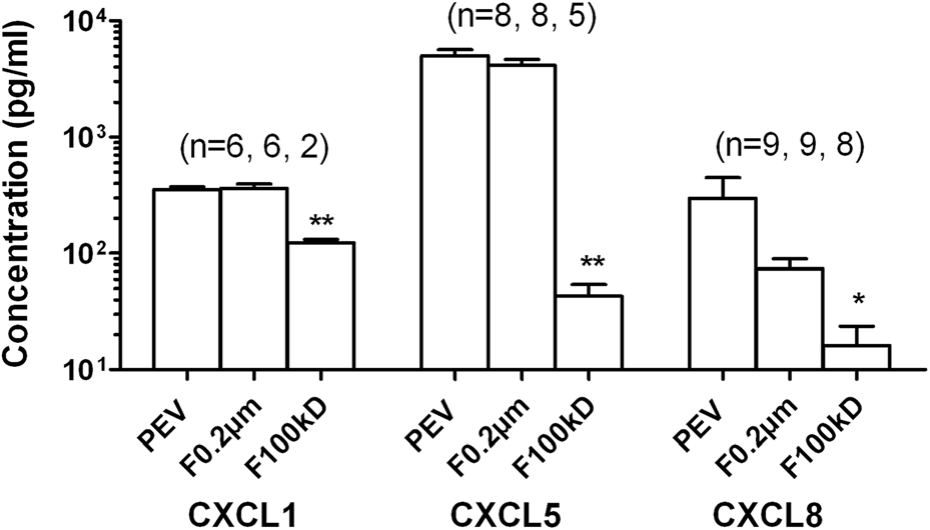
Chemokines detected in vesicles suspensions or filtrates. PEV or filtrates obtained from PEV using 0.2-μm pore filters or 100-kD MW cutoff filters were analyzed by multiplex bead immunoassay measuring CXCL1/GRO-α, CXCL5/ENA-78, CXCL8/IL-8. * = *p* < 0.05, ** = *p* < 0.01 compared to PEV by paired *t* test. Data are mean ± SEM from (*n*) experiments shown above each bar. Not all filtrates were tested in every experiments, but PEV were tested in each experiment.

Analysis of the PEV suspensions by chemokine array revealed presence of the CXC chemokines CXCL1 (GRO-α), CXCL5 (ENA-78), and CXCL8 (IL-8), which operate through CXCR1 and CXCR2 (Fig. 5). CXCL5 was present at high levels (~ 7 ng/ml) in the bioactive range, while CXCL1 and CXCL8 were much lower at about 0.3 ng/ml. To test whether these were associated with PEV, we filtered PEV through 0.2-μm pore or 100-kD MW cutoff column filters. Levels of CXCL5 and CXCL8, but not CXCL1 tended to be reduced by 0.2-μm filters, but this effect was not statistically significant. Each chemokine was reduced after filtration through the 100-kD filter (Fig. 5). Thus, chemokines were borne by a range of sizes of PEV, with a relatively small proportion being soluble and able to pass through the cutoff filter with size much greater than the chemokines themselves (~ 10 kD). These results suggest that PEV borne CD62P and CXC-chemokines played roles in surface-bound PEV-mediated PMN capture and firm adhesion.

### Ability of PEV to Promote PMN Recruitment by HUVEC

**Fig. 6:**
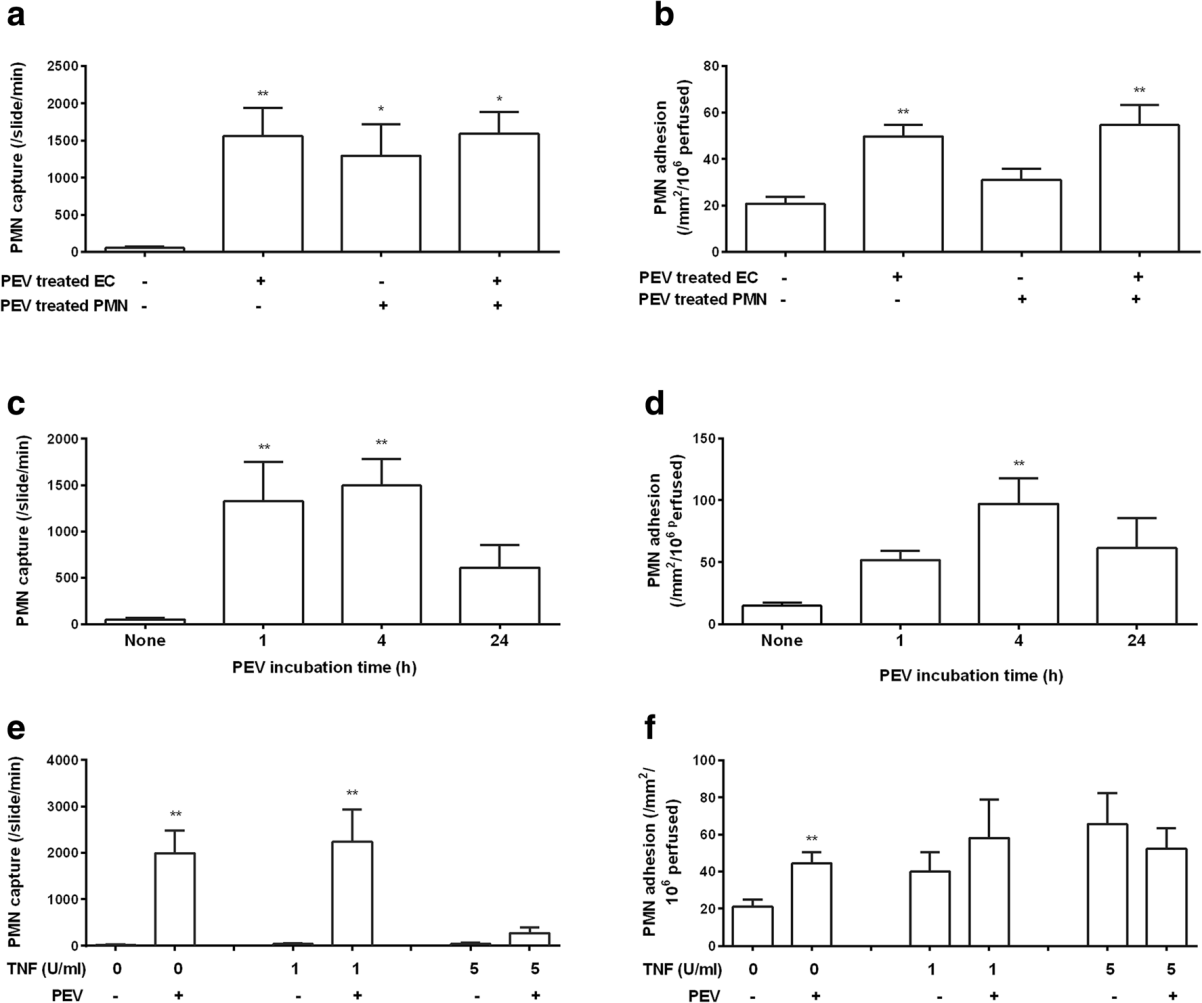
Ability of PEV to promote recruitment of flowing PMN by HUVEC. (**a**) Comparison of the number of transient capture events for PMN perfused at a wall shear stress of 0.5 Pa over untreated HUVEC or HUVEC pre-treated with PEV for 1 h. Neutrophils were perfused alone (*n* = 9) or with PEV added to the cell suspension (*n* = 4). (**b**) Stably adherent PMN on HUVEC after perfusion as above, and washout of non-adherent cells. One-way ANOVA showed a significant effect of treatment combinations on capture events and stable adhesion (*p* < 0.01 in each case). * = *p* < 0.05, ** = *p* < 0.01 compared to untreated EC by Dunnett test. (**c**), (**d**) Effect of varying time of incubation of PEV with HUVEC on transient capture events (**c**) or stable adhesion (**d**) of PMN perfused over HUVEC at wall shear stress of 0.05 Pa (*n* = 6, 11, or 3 for incubation of 1, 4, or 24 h). One-way ANOVA showed significant effect of PEV incubation (*p* < 0.01 in **c**, *p* < 0.05 in **d**). ** = *p* < 0.01 compared to untreated EC by Dunnett test. (**e**), (**f**) Effect of adding PEV to HUVEC treated with different concentrations of TNF for 4 h, on transient capture events (**e**) or stable adhesion (**f**) of PMN perfused over HUVEC at wall shear stress of 0.05 Pa (*n* = 7, 5, 4 for 0, 1, or 5-U/ml TNF). ** = *p* < 0.01 compared to HUVEC without PEV, tested separately at each TNF concentration, by paired *t* test. All the data are mean ± SEM from *n* experiments.

To determine whether PEV could induce adhesion of PMN to HUVEC, we first treated HUVEC with PEV for 1 h and then perfused PMN at a wall shear stress of 0.05 Pa. Video observations demonstrated significant increase in PMN capture from flow compared to untreated HUVEC, which did not support transient attachments (Fig. 6a). PEV-treated HUVEC also supported a greater number of firmly adherent PMN, about twice as many as observed for untreated HUVEC (Fig. 6b). Interestingly, perfusion of PMN along with PEV also caused short-lived attachments on HUVEC which had not themselves been pre-treated with PEV, although this form of treatment did not significantly increase firm adhesion (Fig. 6a, b). Moreover, when treated PMN were perfused over treated HUVEC, levels of capture or firm adhesion was similar or modestly increased compared the values for experiments where only the HUVEC had been treated with PEV (Fig. 6a, b). These results suggest that only a short exposure of HUVEC to PEV is required to cause formation of short-lived attachments, although longer may be required to induce firm adhesion.

Studies of PMN perfused over HUVEC were thus repeated for different periods of incubation of the HUVEC with PEV (1, 4, and 24 h). Pretreatment of HUVEC with PEV for 1 or 4 h caused significant increases in inflow capture, but the number had decreased by 24 h incubation (Fig. 6c). Firm adhesion of PMN increased after 1-h pre-incubation with PEV, increased further at 4 h, and then decreased by 24 h (Fig. 6d). These results support the concept of two phases of response to PEV by ECs, but indicate that over prolonged incubation, capture and stimulation of firm adhesion wane.

We also tested the effects of PEV treatment of HUVEC in the presence of increasing concentrations of TNF. TNF treatment alone did not induce short-lived capture events, but did cause dose-dependent increase in firm adhesion (Fig. 6e, f). PEV caused many transient attachments for HUVEC treated with 0- or 1-U/ml TNF, but few of these events were observed for 5 U/ml (Fig. 6e). PEV increased firm adhesion significantly only for the otherwise untreated HUVEC; at 1 U/ml TNF, there was a slight but not significant increase in adhesion with PEV, but not at 5-U/ml TNF (Fig. 6f). In two experiments with 100-U/ml TNF, transient capture events were not observed with or without PEV, and incubation with PEV did not alter the already very high levels of stable adhesion (data not shown). This data suggests that PEV can initiate PMN recruitment but have little extra effect for EC stimulated by cytokines above a low level.

**Fig. 7:**
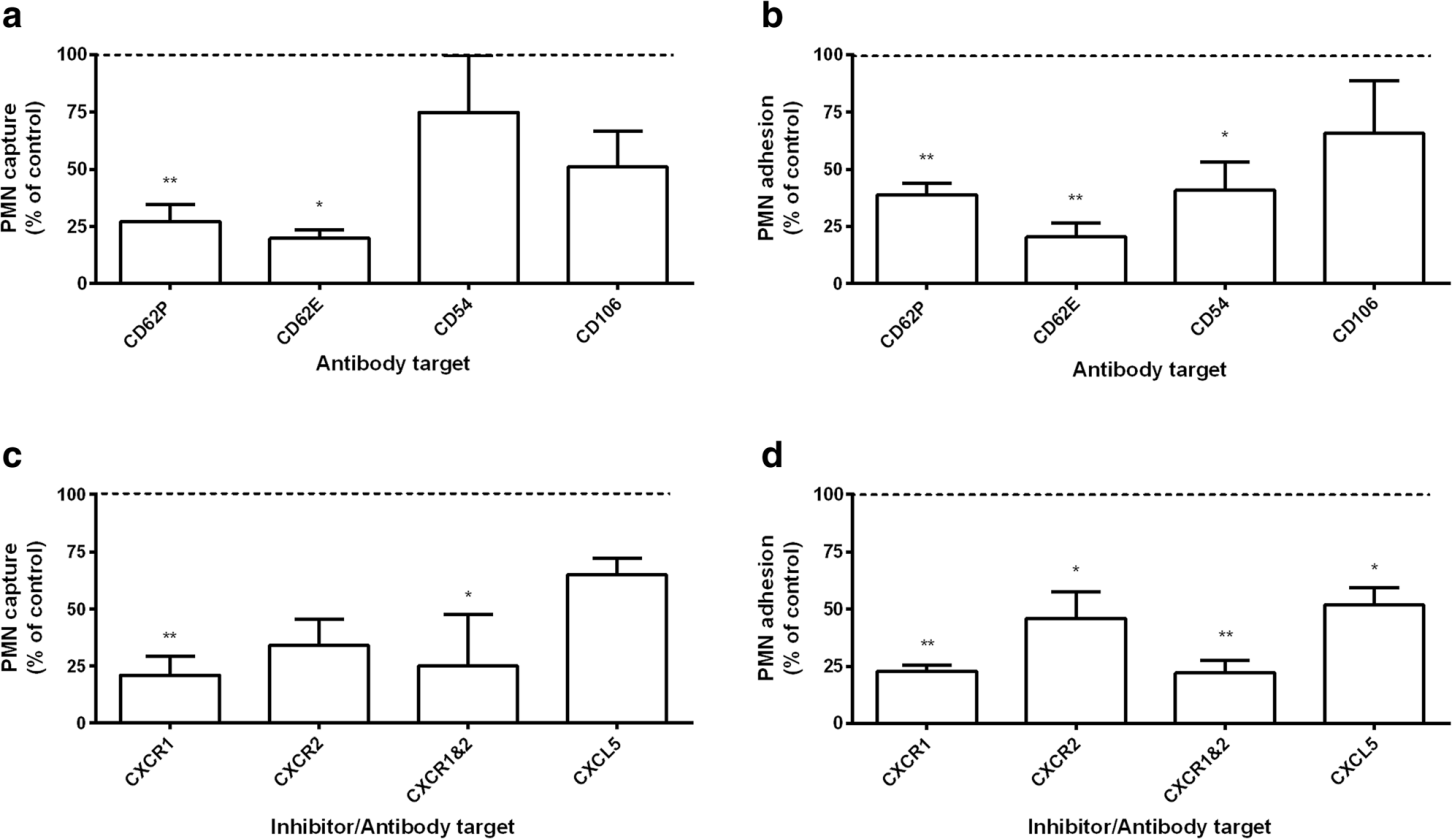
Mechanisms by which PEV promote recruitment of flowing PMN by HUVEC. (**a**), (**b**) Effects of blocking adhesion receptors on HUVEC after incubation for 4 h with PEV, on transient capture events (**a**) or stable adhesion (**b**) of PMN perfused over HUVEC at a wall shear stress of 0.05 Pa. HUVEC were treated with anti-CD62P (*n* = 6), anti-CD62E (*n* = 4), anti-CD54 (*n* = 4), or anti-CD106 (*n* = 4). (**c**), (**d**) Effects of blocking receptors on PMN or chemokine CCL5 on HUVEC on transient capture events (**c**) or stable adhesion (**d**) of PMN perfused over HUVEC treated with PEV for 4 h. PMN were treated with anti-CXCR1, anti-CXCR2, or both (each, *n* = 3), or HUVEC was treated with anti-CXCL5 (*n* = 4). Not all treatments were used in all experiments, but untreated controls were tested in each experiment. All data are mean ± SEM from *n* experiments expressed relative to control without antibodies. * = *p* < 0.05 compared to control by paired *t* test.

Further investigations aimed to dissect the mechanisms promoting PMN interactions with HUVEC treated with PEV for 4 h. Treatment of HUVEC with antibody against CD62P after earlier incubation with PEV reduced transient attachments and stable adhesion by about 80 and 60%, respectively (Fig. 7a, b). Similar levels of inhibition were obtained if the HUVEC were treated with antibody against E-selectin (Fig. 7a, b). Blockade of CD54 (ICAM-1) did not inhibit transient capture but did reduce the level of firm adhesion by 56%, while blockade of CD106 (VCAM-1) did not have significant effects on capture or firm adhesion (Fig. 7a, b). We also tested mechanisms which activated PMN after HUVEC had been treated with PEV. Blockade of CXC receptor 1 or 2 on PMN significantly reduced PMN capture and adhesion by about 75%, with similar effect when both were blocked (Fig. 7c, d). In addition, incubating HUVEC cultures with blocking antibody against CXCL5 reduced PMN firm adhesion by nearly 50%, but did not reduce inflow capture significantly (Fig. 7c, d). Overall, these results suggest that selectins provided or induced by PEV supported capture, while stabilization of adhesion arose from activation through CXC-chemokines.

### Binding and Bioactivity of PEV Sub-populations for PMN

**Fig. 8:**
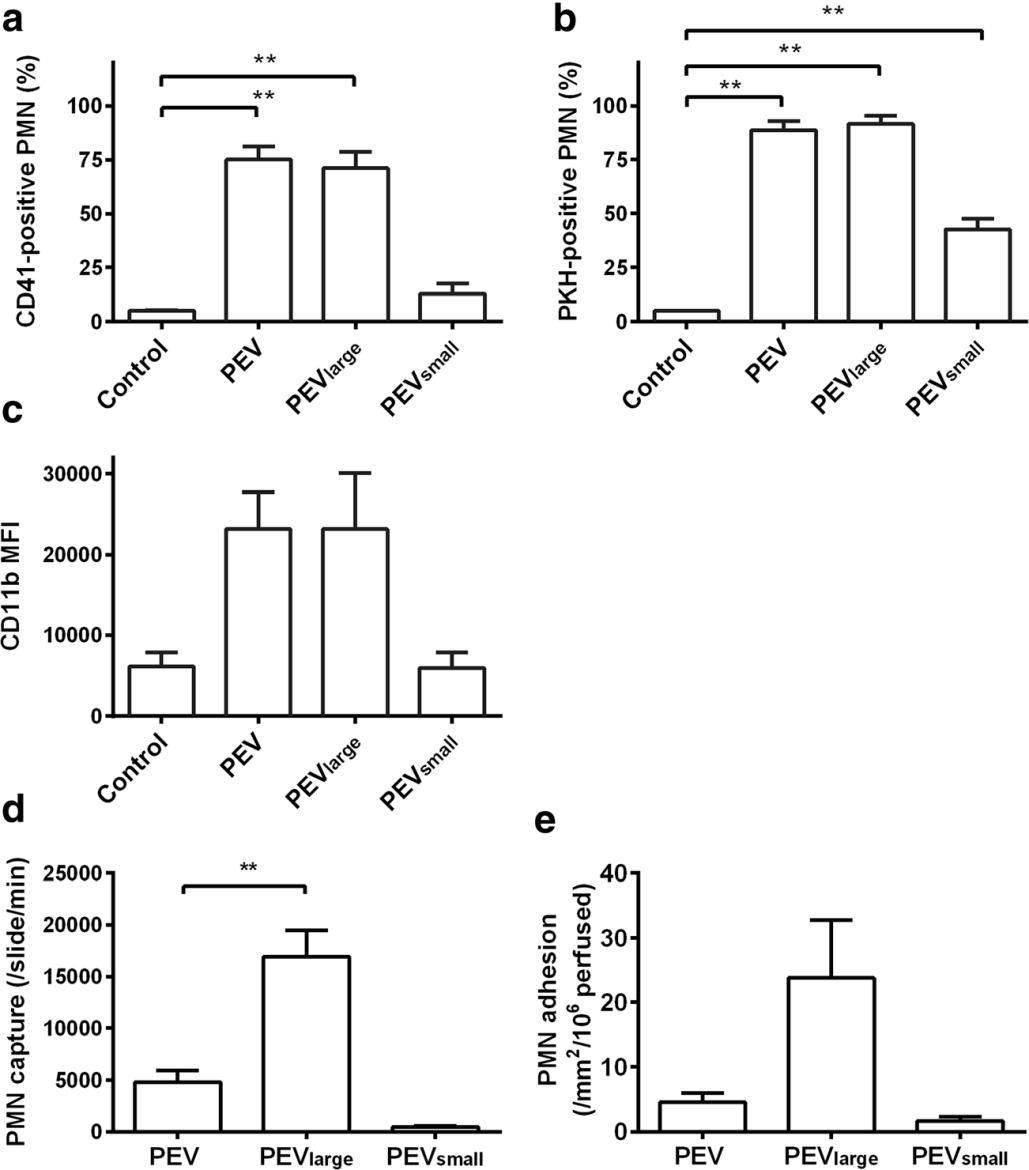
Binding of different vesicles fractions to PMN and ability to support PMN recruitment from flow. (**a**) Percentage of PMN positively stained after incubation for 30 min with PEV, PEV_large_ or PEV_small_ derived from platelets labeled with anti-CD41 (*n* = 5 for all treatments). (**b**) Percentage of PMN positively stained after incubation for 30 min with PEV, PEV_large_, or PEV_small_ derived from platelets labeled with PKH67 (*n* = 3 for all treatments). In (**a**) and (**b**), one-way ANOVA showed significant effects of treatments (*p* < 0.01). ** = *p* < 0.01 compared to control without added PEV by Dunnett test. (**c**) Surface expression of CD11b on PMN after incubation for 30 min with PEV, PEV_large_, or PEV_small_ determined by MFI (*n* = 5 for all treatments). One-way ANOVA showed a significant effect of treatment (*p* < 0.05) and borderline significance compared to control without added PEV by Dunnett test for PEV (*p* = 0.06) and PEV_large_ (*p* = 0.07). **(d**) Transient capture events for PMN perfused at a wall shear stress of 0.1 Pa over surfaces coated with PEV, PEV_large_, or PEV_small_ (*n* = 4 for each surface). One-way ANOVA showed significant effect of treatment (*p* < 0.01). ** = *p* < 0.05 compared to PEV by Dunnett test. (**e**) Stably adherent PMN on surfaces coated with PEV, PEV_large_, or PEV_small_, after perfusion under the same conditions (*n* = 4 for each surface). One-way ANOVA showed borderline significant effect of treatment (*p* = 0.054). All data are mean ± SEM from *n* experiments.

We tested the ability of PEV_large_ or PEV_small_ fractionated from PEV from CD41- or PKH-labeled platelets to bind and label PMN. PEV and PEV_large_ gave similar levels of PMN label for either CD41 or PKH67 (~ 70 or ~90% positively labeled, respectively) (Fig. 8a, b). PEV_small_ on the other hand only yielded ~ 10% labeled PMN for CD41, but ~ 40% for PKH (Fig. 8a, b). The smaller PEV may not carry sufficient CD41 to provide detectable label, but apparently carry enough of the non-specific membrane label to make binding to PMN detectable. We also observed that CD11b expression was increased for PMN incubated with PEV_large_ or PEV, but not with PEV_small_ (Fig. 8c). The different fractions were also coated on microslides, and their ability to directly capture and cause adhesion of PMN was tested. PEV_larg_ were highly effective in capture and also more effective than unfractionated PEV in causing stable adhesion of PMN (Fig. 8d, e). In contract, PEV_small_ were ineffective for either forms of recruitment.

## DISCUSSION

Extracellular vesicles released from activated platelets have been shown to interact with leukocytes and ECs and thereby modulate inflammation [41, 42]. However, their precise roles in promoting capture and activation of flowing PMN by endothelium have not been fully dissected to date. Here, PEV were isolated from platelets activated by collagen-related peptide (CRP-XL) and found to bind efficiently to PMN or to EC. They were also able to activate these cells as evidenced by increased expression of adhesion molecules (*e.g.*, CD11b or ICAM-1, respectively). Interestingly, when PEV were deposited on an inert surface, they were able to directly capture flowing PMN, supporting large numbers of short-lived attachments that were occasionally converted to stable adhesion. Treatment of otherwise-unstimulated EC with PEV also caused appearance of multiple short-lived attachments and an increase in stable attachments, with the stable adhesion more prominent after treatment for 4 h than after 1 h. These effects were still evident if the EC had been treated with a low dose of TNF (1 U/ml), but were not detectable among the high levels of capture and activation of PMN which occurred when the EC were pre-treated with 100 U/ml TNF. PEV preparations could be divided into larger platelet microvesicles and smaller vesicles enriched in exosomes, and soluble agents present in the suspending medium. Separation of the fractions by centrifugation or filtration indicated that the effects of PEV could be attributed largely to the platelet microvesicles, which even carried most of the chemokines found in the PEV preparation.

The number and diameter of vesicles from about 50 nm upwards were quantified by nanoparticle tracking analysis. Their binding to PMN or EC was quantified by flow cytometry, using PEV released by platelets that had been pre-labeled with fluorescent antibody against CD41 (α_IIb_-integrin) or the non-specific membrane dye PKH67. Flow cytometry could only detect the larger vesicles, but showed that the great majority were labeled by these methods and allowed dynamics of the increasing binding to target cells to be investigated. PMN rapidly bound PEV and upregulated their expression of CD11b in a dose- and time-dependent manner. The binding itself was Ca^++^-dependent and attributable largely to CD62P (P-selectin) and stabilized by CD11b. About half of EC became labeled by PEV within 1 h and activation over 4 h was evidenced by increases in expression of adhesion molecules, VCAM-1, ICAM-1, and E-selectin. The binding was again Ca^++^-dependent but not through P-selectin in this case. Several classes of integrins contributed to binding in an RGDS-dependent manner. The activatory responses observed are largely consistent with previous studies of PMN [22] and EC [12] incubated with platelet “microparticles.” P-selectin has been widely shown to support interaction of PMN with platelets [35, 39, 43], although it appeared to be a minor player in interactions with PEV in one study [12]. Mechanisms of adhesion of PEV to ECs have not been described before to our knowledge, although the platelet αvβ3-integrin receptor has been implicated in RGDS-dependent binding of intact platelets to HUVEC [44].

The ability of PEV to directly capture flowing leukocytes has not been described before. The interactions with PMN, although transient, resulted in the firm adhesion of a small proportion of the cells perfused at a wall shear stress of 0.1 Pa. The interactions were greatly reduced if deposited PEV were treated with antibody against P-selectin. Decreasing the shear stress from 0.1 Pa resulted in increased rates of capture, but more evidently, in the numbers becoming firmly adherent. Forlow et al. [12] demonstrated that platelet microparticles induced an increase in interaction between flowing leukocytes and other surface-bound leukocytes through P-selectin, but did not study capture by PEV alone. If PEV were added to PMN and perfused over PEV-coated surfaces, capture and especially stable adhesion were increased over levels for PMN perfused alone over the coated surface. This was likely due to the activation of the perfused PMN by the addition of PEV, thus increasing their firm adhesion to the deposited PEV.

We also tested if PEV could directly mediate PMN recruitment on HUVEC from flow, and/or whether prolonged incubation could have an indirect effect through endothelial activation. We observed that PEV promoted the transient capture of flowing PMN by otherwise unstimulated EC. If PEV were added only to perfused PMN, capture was as effective as if the EC had been pre-treated with PEV. Indeed, pretreatment of EC for 1 or 4 h had similar effects on capture from flow. Thus, it appears that PEV can quickly induce capture events without regulation of endothelial receptors. Longer 4-h incubations of EC with the PEV did increase stable adhesion of PMN, suggesting activation of the EC. After 24-h incubation, capture events had decreased markedly and stable adhesion also, suggesting that surface presented receptors from the PEV had been lost or absorbed, and the activatory response had waned. Interestingly, antibody blocking studies showed roles for CD62E (E-selectin) as well as P-selectin in capture from flow, supporting the concept of a dual role for direct (P-selectin) and indirect (E-selectin upregulation) effects of PEV after prolonged treatment of EC. Stable adhesion required the selectin-mediated capture and was itself supported by CD54 (ICAM-1), which we had earlier demonstrated to be upregulated by PE by flow cytometry.

Apart from supplying P-selectin to promote capture, the PEV had stimulatory capacity for PMN. Indeed, platelets are a rich source of chemokines, and of these, PEV have been shown to deliver CXCL5 (ENA-78) at least [45–47]. Blockade of CXCR1 and CXCR2 on PMN inhibited firm adhesion, but not capture, on surface bound PEV, suggesting that PMN activation occurred through these receptors. We thus screened PEV for CXCL1, 5 and 8. All were detectable, with CXCL5 having the highest concentration. Passage of PEV suspension through a 100-kD cutoff filter greatly reduced concentrations of the chemokines. Since their molecular weights are ~ 10 kD, they were mainly associated with the vesicles rather than in solution. Filtration though 0.2-μm pore filters did not reduce levels of CXCL1, but did reduce CXCL5 and CXCL8, indicating that the larger PMV carried a proportion of the latter chemokines at least. However, it should be noted that nanoparticle tracking analysis indicated that > 90% of vesicles were below 0.2 μm in diameter, and these vesicles could clearly carry significant levels of chemokines.

PMN capture could also be transformed to firm adhesion on EC which had been treated for PEV for 4 h. In this case, treatment of PMN with function-blocking antibodies against CXCR1 or CXCR2 or both decreased capture and stable adhesion of the PMN. Blocking CXCL5 (the chemokine found with highest concentration in PEV) on the EC had a non-significant effect on capture but significantly reduced stable adhesion. The conversion to stable adhesion may have been through CXCL5 transferred to EC by PEV, as well as chemokines generated by EC after activation by PEV. The effect of blocking the chemokine receptors on PMN on the transient capture events was not expected, as no ability to support initial adhesion has been reported for these receptors before. When EC were treated with increasing concentrations of TNF along with PEV, the effects of the PEV on capture and stable adhesion decreased. Thus, transfer of receptors or activating agents to the EC became outweighed by the response of the EC to TNF, which would induce increasing upregulation of selectins, CXC chemokines, and ICAM-1 [48, 49].

Our study also gave insight into the relative bioactivities of the vesicular constituents of PEV. When PMV and Pexo were enriched by centrifugal methods, PMV were able to transfer much higher levels of CD41 to PMN than the fraction enriched with exosomes. They also transferred more of the non-specific membrane label PKH67, although the small vesicle fraction did transfer this label as well. It was also notable that only larger vesicles activated PMN to upregulate CD11b expression, and when coated on a surface, only they were able to support capture and stable adhesion of flowing PMN. Thus, while both PMV and Pexo could bind to PMN, transfer of surface receptors was more effective for larger vesicles, and the ability to directly induce PMN activation or support recruitment from flow was restricted to them. Proteomic analysis has previously demonstrated that PMV and Pexo have different composition and content of platelet markers [34]. However, we are not aware of studies of their differential effects on leukocytes.

In summary, we have demonstrated that PEV can directly activate PMN and also support a two-step recruitment process through delivery of selectins and chemokines to a surface. PEV can also activate EC to promote PMN adhesion. Although this response is not as great as that induced by high concentrations of TNF, it may potentiate lower level stimuli. Generation of PEV is associated with a range of thrombotic and inflammatory pathologies [5–7], and our results support the concept that their levels are not only markers of events, but will also influence their outcome.
